# Implementing a care pathway for elderly patients, a comparative qualitative process evaluation in primary care

**DOI:** 10.1186/s12913-015-0751-1

**Published:** 2015-03-04

**Authors:** Tove Røsstad, Helge Garåsen, Aslak Steinsbekk, Erna Håland, Line Kristoffersen, Anders Grimsmo

**Affiliations:** Department of Public Health and General Practice, Norwegian University of Science and Technology (NTNU), Trondheim, Norway; Department of Health and Welfare Services, City of Trondheim, Trondheim, Norway; Department of Adult learning and Counselling, Norwegian University of Science and Technology (NTNU), Trondheim, Norway; Norwegian Health Net, Trondheim, Norway

**Keywords:** Care pathway, Continuity of patient care, Checklists, Primary care, Home care services, Implementation, Complex intervention, Process evaluation, Health care quality, Normalization Process Theory

## Abstract

**Background:**

In Central Norway a generic care pathway was developed in collaboration between general hospitals and primary care with the intention of implementing it into everyday practice. The care pathway targeted elderly patients who were in need of home care services after discharge from hospital. The aim of the present study was to investigate the implementation process of the care pathway by comparing the experiences of health care professionals and managers in home care services between the participating municipalities.

**Methods:**

This was a qualitative comparative process evaluation using data from individual and focus group interviews. The Normalization Process Theory, which provides a framework for understanding how a new intervention becomes part of normal practice, was applied in our analysis.

**Results:**

In all of the municipalities there were expectations that the generic care pathway would improve care coordination and quality of follow-up, but a substantial amount of work was needed to make the regular home care staff understand how to use the care pathway. Other factors of importance for successful implementation were involvement of the executive municipal management, strong managerial focus on creating engagement and commitment among all professional groups, practical facilitation of work processes, and a stable organisation without major competing priorities. At the end of the project period, the pathway was integrated in daily practice in two of the six municipalities. In these municipalities the care pathway was found to have the potential of structuring the provision of home care services and collaboration with the GPs, and serving as a management tool to effect change and improve knowledge and skills.

**Conclusion:**

The generic care pathway for elderly patients has a potential of improving follow-up in primary care by meeting professional and managerial needs for improved quality of care, as well as more efficient organisation of home care services. However, implementation of this complex intervention in full-time running organisations was demanding and required comprehensive and prolonged efforts in all levels of the organisation. Studies on implementation of such complex interventions should therefore have a long follow-up time to identify whether the intervention becomes integrated into everyday practice.

## Background

The complexity of elderly patients’ health situation requires more coordinated health care across health care levels than what is currently offered, especially in the transitional phase between hospital discharge and primary care [1-3]. Several strategies, including a range of interventions, have been developed to improve continuity of care across care levels; e.g. individualised discharge planning [4], liaison nurses and discharge coordinators [5], enhanced multidisciplinary team work [6], transitional and intermediate care units [7], integrated care pathways [8] and integrated medical and social care [9]. These are complex interventions including multiple components and personnel, often across different organisations and care levels. A successful implementation may be crucial for the effect. Thorough analysis of the implementation process is therefore called for when introducing new interventions [10].

**Figure 1:**
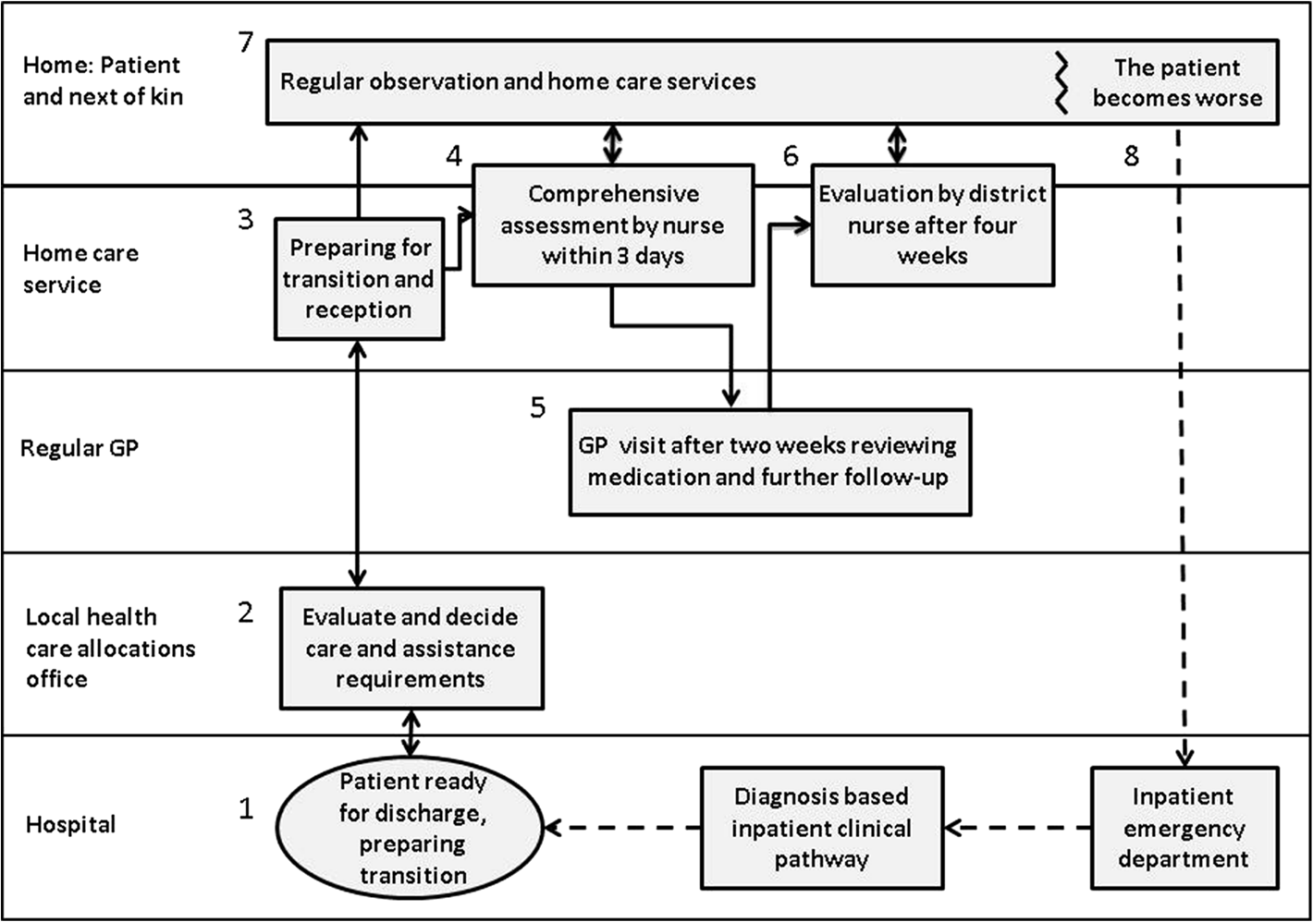
**Generic care pathway (PaTH), for transition from hospital and follow-up of home care recipients** **[** 2 **]** **.** The boxes represent procedures and checklists and the arrows the flow of information between involved parties. It starts with the patient being reported ready for discharge and information is exchanged (1, 2 and 3). Within three days a home care nurse performs a thorough and structured assessment (4). The patient has a consultation with the GP 14 days after discharge (5), and a nurse or nursing assistant performs an extended assessment during the first four weeks (6). A daily care plan is continuously updated (7), and if the patient’s condition gets worse, the home care service has a routine for what to observe, whom to contact and which information to pass on (8). The checklists included practical issues (e.g. whether assistive devices had been ordered and when they would be installed), health issues (e.g. review of medication), social conditions (e.g. if the present accommodation was appropriate for the patients’ level of functioning) and physical and cognitive functioning (e.g. ability to climb stairs, reduced memory). Some checklists were to be used by nurses only (3 and 4), while others were also to be used by nursing assistants (6 and 8). All of the issues on the lists were not necessarily relevant for all patients and the nurses and nursing assistants had to use their professional insight to decide what to assess and how to follow-up.

In 2009, a generic care pathway (**Pa**tient **T**rajectory for **H**ome-dwelling elders – PaTH, Figure 1), intended to improve continuity of care and reduce the need of institutional care, was developed and introduced in six municipalities in Central Norway. PaTH was the result of a bottom-up process in which home care professionals, general practitioners, patient organisations, and hospital employees (nurses and physicians) defined challenges and proposed solutions in transitional care and follow-up [2].

The aim of this study was to investigate the process of implementing PaTH into everyday practice by comparing the joint experiences of health care professionals and managers in home care services between the municipalities where it had been introduced.

## Methods

### Design

This was a qualitative study where the process of implementing PaTH was compared in six municipalities through individual and focus group interviews of leaders and regular staff, supported by reflexive field notes and minutes from meetings.

### Setting, informants and ethics

In Norway health care and social care services are universally accessible, and are mainly financed by and provided within the public sector [11]. Local authorities (municipalities), the lowest level of public administration, are responsible for providing primary health care, including home care and medical services [11] (Table 1).

**Table 1:** **Ambulant home care services and general practices in six Norwegian municipalities (A-F) introducing PaTH**

**Information about the municipalities**	**A**	**B**	**C**	**D**	**E**	**F**
	**City**	**Rural area**	**Small town**	**Rural area**	**Rural area**	**Rural area**
**Inhabitants**	180 000	6000	11 000	4000	7000	10 000
**Home care recipients** ^**1**^	3000	160	350	170	300	200
**Home care units** ^**2**^	12	1	1	1	1	1
**Home care managers** ^**3**^	12	1	1	1	1	1
**Head nurses** ^**4**^	12	1	2	1	5	3
**Regular staff** ^**5**^	337	24	42	29	53	28
**General practices**	38	1	2	1	2	2
**General practitioners (GPs)** ^**6**^	140	6	8	4	6	7

^1^Persons who receive health and social care because of reduced functional level. Care may be provided several times a day and at night in their own homes.

^2^Every municipality has one or more home care units, which are divided in teams serving the population in a geographical area.

^3^Responsible for economy, personnel and quality in home care services.

^4^Responsible for daily professional activities, including guidance and supervision of staff.

^5^Includes nurses and nursing assistants. The numbers refer to full-time equivalents.

^6^Medical services to home-dwelling inhabitants are delivered by GPs who usually work in group practices. GPs operate independently of the home care services. Due to the inhabitants’ right of free choice of a regular GP, the GPs may have patients in common with all home care units in the municipality where they work and also in home care units in neighbouring municipalities.

PaTH was introduced in the municipalities in the period October 2009 – March 2010. All home care staff received detailed instruction about PaTH at the time of introduction in the form of a one-day course where four of the authors of the present article (TR, LK, AG and HG) gave lectures during the introduction course. The home care managers were responsible for further training in the home care units. To monitor progress of the implementation of PaTH, TR had monthly conference calls with the head nurses or home care managers in each of the municipalities. LK was a manager in one of the participating municipalities, but was not involved in the interviews.

All other home care managers and all head nurses in the home care units that introduced PaTH participated in the interviews in the current study. Furthermore, they recruited regular staff (nurses and nursing assistants) who had worked in home care since the introduction of PaTH.

Home care managers received written information about the study before the interviews. TR explained the purpose of the study to all the informants, that citations would be anonymous, that they could ask for statements to be deleted, and that the interviews would be handled confidentially. All informants signed an informed consent document before participation. The study was approved by the Regional Committee for Medical and Health Research Ethics in Central Norway and the Ombudsman for Research and Social Science Data Service.

### Data collection

Focus group interviews and individual interviews were the main data sources. Managers and head nurses from all home care units participated in two focus group interviews in November 2011, 20 – 25 months after the introduction of PaTH (Table 2). The other focus group interviews took place in each municipality from March 2012 to February 2013 and included regular staff as well as the management level. Management and regular staff were interviewed separately for the informants to speak more freely. TR led all interviews and co-author EH participated in the two last focus group interviews.

**Table 2:** **The number and type of interviews and informants by year**

**Year**	**Type of informants**	**Number of focus group interviews**	**Number of individual interviews**	**Total number of informants**
**2011** ^**1**^	Home care managers and head nurses	2	0	13
**2012** ^**2**^	Home care managers and head nurses	2	2	7
	Nurses and nursing assistants	6	0	26
**2013** ^**2**^	Home care managers	1	0	6
	Nurses and nursing assistants	1	0	8
**Total**		12	2	60

^1^Focus group interviews with representatives from all municipalities in November 2011.

^2^Focus group interviews and individual interviews in every municipality March 2012 – January 2013.

A semi-structured interview guide, used during the interviews, included the following topics: how the informants had been involved with the care pathway, their initial expectations, how it had been introduced at their workplace, the efforts invested to take it into use, challenges, promoting factors, assessments of benefits, and if and why it was dismissed or integrated and sustained in daily use.

Data sources, in addition to interviews, were minutes from the monthly conference calls with the head nurses or home care managers during the first year. Furthermore, TR made reflexive field notes on the overall impression of the implementation process when visiting the municipalities in 2012. The field notes were based on the interviews, assessments on how PaTH was integrated in the electronic health records, and informal discussions with the home care managers.

### Theoretical framework

Among the many different frameworks used in implementation studies [12-16], we chose the Normalization Process Theory (NPT) to guide our analyses, as it offers a framework for evaluation of complex interventions and for comparing the implementation processes across different sites [17-19]. It helps to explain the processes by which complex interventions become, or do not become, integrated in everyday health care practice (i.e. ‘is normalised’) which was the ambition when introducing PaTH [20]. NPT has four core constructs which are all seen as essential for new working practices to become a natural part of daily work: *coherence* (making tasks meaningful and understandable), *cognitive participation* (building commitment and engagement), *collective action* (efforts and resources invested to make the intervention function), and *reflexive monitoring* (assessment of benefit).

### Data analyses

The interviews were audio-recorded and transcribed verbatim, checked, anonymised, and corrected against the audio files by the first author (TR). In accordance with Malterud’s method for systematic text condensation [21], all the authors independently first read all the interviews to get an overview of the material and to identify preliminary themes associated with implementation of PaTH. The preliminary themes were first discussed by the authors TR, EH, HG and LK. TR identified ‘meaning units’ that were classified into themes and subthemes. These were subsequently refined through discussions among all the authors in an iterative process. TR wrote a summary of the subtheme contents and identified illustrative quotations. In the last step, the NPT framework was used to map the themes to facilitate a systematic comparison between the municipalities. The comparison is based on what was perceived to be the common understanding of the implementation process among the informants from each municipality 24 or 32 months after PaTH was introduced. Minutes from conference calls together with the reflexive field notes, supplemented the analyses of the interviews.

TR and EH re-read the interviews, field notes, and the minutes after the analyses to validate whether the synthesis and illustrative quotations still reflected the original context appropriately. The results were presented to the home care managers in all municipalities for identification of any apparent misunderstandings. A few details on value for managers and user friendliness in one municipality were commented on and subsequently corrected.

## Results

Home care professionals in all municipalities used PaTH when interviewed in 2011. At the time of the interviews in 2012/2013, PaTH was used in full scale in daily work in municipalities A and B (Table 3). Some elements of PaTH was used in two municipalities (C and D), but occasionally and not by all staff, and in the last two municipalities (E and F) PaTH was discontinued. Key themes and subthemes of importance for the implementation process, mapped onto the four main constructs of NPT, are summarised in Table 3 and are further detailed below.

**Table 3:** **Differences in implementation status and implementation process in six municipalities (A-F)**

	**Municipalities**					
	**A**	**B**	**C**	**D**	**E**	**F**
	**PaTH in use in full scale** ^**1**^		**Elements of PaTH in use** ^**1**^		**PaTH not in use** ^**1**^	
**Makes sense (coherence** ^**2**^ **)**						
Expecting PaTH to be useful	Yes	Yes	Yes	Yes	Yes	Yes
Regular staff understood how to use PaTH	Mixed	Mixed	Mixed	Mixed	Mixed	Mixed
**Commitment and engagement (cognitive participation** ^**2**^ **)**						
Sustained leadership	Yes	Yes	No	No	No	No
Practice in using checklists	Intensive	Intensive	Minimal	Minimal	Minimal	Minimal
General attention to PaTH at workplace	Yes	Yes	No	Nurses only	No	No
**Facilitating use of PaTH (collective action** ^**2**^ **)**						
Extra personnel resources	Yes	Yes	No	Yes	No	No
Major competing priorities	No	No	No	No	Yes	Yes
Usability in electronic health record	Good	Fair	Poor	Poor	Poor	Poor
Working schedule facilitated for PaTH	Yes	Yes	No	No	No	No
Checklists incorporated in daily routines	Yes	Yes	No	No	No	No
**Value of PaTH (reflexive monitoring** ^**2**^ **)**						
Impact on collaboration with the hospital	Mixed	Mixed	No	No	No	No
Impact on collaboration with GPs	Yes	Yes	No	Yes	No	No
Impact on service quality	Yes	Yes	No	Yes	No	Yes
Value for individual nurse/nursing assistant	Yes	Yes	No	No	No	No
Valued as a management tool	Yes	Yes	No	Yes	No	No

^1^Assessed 24 months (B-F) and 32 months (A) after introduction of PaTH in the municipalities.

^2^Core constructs of the Normalization Process Theory.

### Makes sense

As home care professionals in all participating municipalities had been involved in development of PaTH according to their own perceived needs for improvements, informants from all municipalities expected PaTH to be useful; i.e. to improve collaboration with GPs and hospitals and the quality of service delivery within the home care services. However, the process of creating a collective understanding of responsibilities and how to use PaTH was found to be more demanding than expected:

*A lot of people seem to have trouble understanding what actually has to be done. There is an enormous need for guidance. At first we thought it had been understood and would be used, but…. Experience has shown that an awful lot of supervision and guidance is needed so that they really understand the how’s and why’s of the pathway. (Head nurse, municipality A)*

The main challenge was said to be uncertainty regarding how to observe, assess, act, and document issues on the checklists; especially for nursing assistants who were facing new roles and responsibilities. Their traditional role was mainly to assist patients with practical issues, so they were not familiar with systematic observations, assessments, and documentation of health and functional issues. Some municipalities did not succeed with involving nursing assistants or did not prioritise it.

### Engagement and commitment

Some home care managers underlined the necessity of sustained strong leadership in building and maintaining engagement, understanding and commitment of PaTH:

*We’re not only introducing checklists but also changing the way we think and the way we do things. We have to change our habits, which means that we have to think long-term.* (Home care manager, municipality F)

The head nurses who were expected to drive the implementation work, all described this as very laborious and time consuming. Involving the regular staff was especially difficult in municipalities C-F due to unexpected loss of key personnel, too much work for the head nurses, or too little support from the home care managers:

*We were pretty pushed for time and to make matters worse this came on top of everything else. Maybe the checklists weren’t given priority. Then we just have to fit it in when we can. It’s frustrating when you have to fit it in between everything else. (Head nurse, Municipality C)*

Engagement and commitment was clearly affected by the attention given to PaTH at the workplaces. All informants said they received individual guidance when needed, but informants from three municipalities (C, E and F) were not able to recall any general attention to PaTH at the workplace after the introduction course. In municipality D, PaTH was discussed only at nurse meetings. Informants from the municipalities A and B said that PaTH was on the agenda in all common meetings at the work place, and that it was referred to in many other settings; e.g. discussions of complicated patient cases, unwanted incidents, and collaboration with the hospital and GPs:

*We have to keep it in focus and I make sure that it is an issue in all of our meetings. The only way to ensure that people really understand is to continuously repeat yourself. I try to point out, by using examples, how negative the consequences may be if you don’t use the checklists, how much extra work it can mean. (Head nurse, Municipality A)*

The amount of practice with using PaTH checklists was also considered important. Informants from four of the municipalities (C-F) were only able to recall having used a checklist once or twice themselves and never really got used to them:

*We were more optimistic in the beginning. We were going to manage this! But motivation waned as we didn’t use the checklists very often. I think it would have been better if we had used them a lot straight away so that we could have gotten used to them and had them at our fingertips. (Head nurse, municipality E)*

In the municipalities A and B, management decided that to get practice, all staff were to use the checklists both for patients discharged from hospital and for all other home care recipients. Therefore, the staff got much more training in using and understanding the elements of the checklists than at the other sites, and in particular nursing assistants were involved to a greater extent. In these municipalities the main effort during the first year was to get the staff to use the checklists and familiarise themselves with them. Later, the focus was shifted to the content and the quality of documentation related to the checklists. The informants from these municipalities found that the quality of assessments and measures improved over time.

### Facilitating use of PaTH

In municipalities A, B and D the executive municipal management was said to be a driving force by setting clear requirements for the implementation and supplying extra personnel resources to facilitate implementation of the care pathway and guide the staff. In municipalities A and D support was provided at the administrative level, while in municipality B extra personnel was provided in the home care unit. In municipalities E and F, the implementation work was complicated by concomitant economic cutbacks and major reorganisations in the municipalities.

Efforts made to ensure usability of PaTH in daily working practices differed between the municipalities. The checklists were incorporated into electronic health records (EHR) in all of the municipalities, but accessibility of PaTH in the health records varied:

*The check lists were in the wrong place in the health records and it took ages to find them when you needed to use them. (Nurse, municipality E)*

In municipality A the informants described a system where the templates in the EHR were adapted to the checklists in PaTH. This made the checklists easy to find and complete. The informants considered such facilitation as important for the success of the implementation.

Facilitating the working schedule to PaTH was also considered crucial for implementing the care pathway in daily working practices. In municipalities C-F the use of PaTH was simply added on top of the normal workload. The individual staff member had to create space for this extra work by asking their colleagues to take over some of their other tasks. In municipalities A and B dedicated personnel were responsible for creating space on the task lists for all staff:

*Initially we had to make time ourselves to be able to do it. That was a bit frustrating. We had to organise things in the morning and ask colleagues to take over some of our patients to make time. That caused contention because they already felt that they had more than enough to do. But now we only have to let the people who allocate duties know and they sort it out. It works well now. (Nurse, municipality B)*

Nurses and nursing assistants in municipalities A and B, who had more experience with the checklists than in the other municipalities, found ways to further incorporate the use of checklists in daily routines. The informants said that they had first slavishly gone through each item on the checklist during one home visit. They found this to be excessively time-consuming, and eventually changed their approach. They found that several issues could just as easily be observed while they were providing their normal services. Furthermore, items in some of the checklists could be evaluated over subsequent visits. This way the checklists were more naturally incorporated into daily routines and were perceived as less of a burden.

### Value of PaTH

Opinions as to the benefits of PaTH differed. In municipality E the primary objective of implementing PaTH had been to achieve improved collaboration with the hospital and GPs. Their motivation to use PaTH waned when they experienced that the hospitals and GPs showed little or no interest in the new, agreed procedures:

*Collaboration with the hospital [about the care pathway] didn’t really get started. The hospital never had the information we asked about, they hadn’t collected it. That was desperately frustrating. We began to lose faith and we felt it might not be worth the effort. We felt that it [the pathway] had just become an obstacle. (Head nurse, municipality E)*

A lack of awareness within the hospitals was reported by all the informants, and this was not found to improve. However, some informants said that they still felt that they managed to get more relevant information during transition between care levels as they kept on insisting on being given information in accordance with the agreed procedures in PaTH. They found this to save them considerable work later and they experienced that unwanted incidents during transition, especially related to medication errors, were reduced.

In municipalities A, B and D informants reported that both collaboration and the exchange of information with GPs had improved; in the other municipalities informants reported that this was unchanged.

Informants from municipalities A, B, D and F reported that PaTH had an impact on the service quality: The new routines and use of the checklists made them more observant and helped them to a greater extent to detect and prevent potential problems to their home care recipients:

*We are more on the ball now and pick things up much earlier than before. And because of that we are better at intervening earlier so that we avoid people being admitted to hospital. (Nurse, municipality B)*

In the two municipalities were PaTH was integrated in daily working practices (A and B), PaTH was found to be valuable both for the regular staff and the managers. The informants said they felt that their professional standard had been raised and that their jobs were now more interesting. The home care managers found PaTH useful for getting an overview of skills and needs for guidance among the staff. As staff in home care services work alone in the patients’ homes, their professional competence in observing, assessing, and documenting health issues had been difficult to evaluate. Now, the managers were able to uncover individual and collective strengths and weaknesses to a greater extent by checking individual patient assessments through the structured documentation in the EHR. This helped them to better adapt individual and collective training and guidance to actual needs and thus improve knowledge and skills. Furthermore, PaTH was valued as a useful management tool to achieve a more efficient organisation:

*A consequence of PaTH is that the unit is now well organised. Peoples’ responsibilities are clearer. This has reduced the number of discrepancies and quality is better. The unit now works much more like a piece of well-oiled machinery. (Home care manager, group A)*

## Discussion

The municipalities that gave the implementation high and persistent priority within all core constructs of NPT succeeded in incorporating the care pathway in daily working practices; i.e. two of the six municipalities in our study. The implementation of PaTH was found to be demanding and the amount of work needed for successful implementation generally underrated; the two municipalities that experienced major competing priorities during the implementation period ended up discontinuing PaTH despite initial enthusiasm and high expectations. The factors that most clearly differentiated the municipalities from each other were strong management focus on creating engagement and commitment and on practical facilitation for use of PaTH. However, the study demonstrated that all factors identified to embed the new practice mutually influenced each other: When work processes were facilitated (collective action) and intensive work was invested to ensure that all employees gained experience with the checklists (cognitive participation), the employees got a better understanding of their roles and responsibilities and how to use the tool (coherence) and found a way to incorporate the checklist in daily work (collective action). Furthermore, by getting more experience both managers and regular staff found that the new procedures were useful for the patients, the individual professionals, and the organisation of services (reflexive monitoring). This increased motivation, engagement, and commitment both at the staff and management levels (coherence and cognitive participation).

This non-linear relationship and dynamic interplay of factors in NPT was underlined by May and Finch when they presented the theory [17]. Still, studies applying NPT often highlight issues within one of the constructs as the most important challenges or crucial drivers in the implementation [22-25]. Lack of coherence is often pointed to as an important challenge to implementation processes; the intervention does not make sense or is met with conflicting attitudes [22,23]. Implementation studies on integrated care models from France and Sweden [26,27], argue that for new care models to be accepted, integrated, and sustained in daily work, they must be experienced as effectively dealing with real problems in everyday practice. The same studies conclude that bottom-up processes with collaborating parties, as in our study, are effective in developing such care models.

Our study shows that bottom-up processes and enthusiasm is not enough. Complex interventions like PaTH also have to be actively supported by the management levels to be implemented in the organisation in a sustainable manner. This is supported by other implementation studies [27,28] which find that active involvement by the executive management can be crucial to achieve organisational change, not least to ensure that the intervention matches strategic and organisational priorities. Furthermore, support from the executive level signalises the importance and prestige of the work [27].

For new procedures or organisational change to be embedded and sustained, it must be experienced as useful [29,30]. In the municipalities where PaTH sustained, it was valued both by the regular staff and the managers as a means to raise professional standards and organisation of care. PaTH was thus found to have a potential of fusing professional and managerial concerns in primary care. A study from the UK found, correspondingly, that when care pathways were successfully implemented in hospitals, both managerial and professional needs were met; the care pathways provided a means for managers to better plan and evaluate care processes while the patient-centred focus was valued by professionals [31].

### Methodological strengths and limitations

The main strength of this study was the number of informants and that interviews were done in several rounds and up to three years later. This allowed for a thorough investigation of the feasibility and sustainability of the care pathway. The last interviews were carried out two and three years after the introduction of PaTH, which may increase the risk of recall bias from the early introduction phase. The interviews were, however, supplemented by minutes from conference calls during the first year to ensure valid results.

The selection procedure of regular staff informants by the head nurses or home care managers had an inherent risk of ending up with those who were most positive. We found no indications that this was the case, as the staff advocated both frustrations and enthusiasm. One member of the research group (LK) managed one of the home care units in the project. To avoid bias, LK did not take part in the data collection and several researchers with different professional backgrounds analysed the data. LK’s participation in the research group was considered to strengthen the analysis as she had detailed understanding of the context of the home care services. The findings were also validated by presenting them to and getting approval from all the home care managers in all municipalities.

We consider findings regarding the implementation process to be valid beyond our study, as the identified factors were recognisable from other studies and within the theoretical framework of NPT, a theory that has proved to be valid in different contexts in other countries [19]. PaTH itself may however not be a feasible care pathway in countries other than Norway, as health care is organised differently.

### Implications of findings

Our findings illustrate how a comparative process evaluation and use of the NPT framework may help to identify hindrances and facilitators in the implementation process. This is important both to understand the implementation process, to assess the implementation potential before deciding on further deployment of the care pathway and to identify contextual factors of importance when evaluating the effect in a randomised controlled trial [30,32].

## Conclusion

The generic care pathway for elderly patients has a potential of improving follow-up in primary care by meeting professional and managerial needs for improved quality of care, as well as more efficient organisation of home care services. However, implementation of this complex intervention in full-time running organisations was demanding and required comprehensive and prolonged efforts in all levels of the organisation. Studies on implementation of such complex intervention should therefore have a long follow-up time to identify whether the intervention becomes integrated in everyday practice.

## Abbreviations

EHR: Electronic health record
GP: General practitioner
NPT: Normalization process theory
PaTH: Patient trajectory for home–dwelling elders
UK: United Kingdom
